# Rural–Urban and Racial/Ethnic Disparities in Invasive Cervical Cancer Incidence in the United States, 2010–2014

**DOI:** 10.5888/pcd16.180447

**Published:** 2019-06-06

**Authors:** Lulu Yu, Susan A. Sabatino, Mary C. White

**Affiliations:** 1 Washington University School of Medicine, Saint Louis, Missouri; 2 Centers for Disease Control and Prevention, National Center for Chronic Disease Prevention and Health Promotion, Division of Cancer Prevention and Control, Atlanta, Georgia

## Abstract

**Introduction:**

Racial and socioeconomic disparities exist in cervical cancer screening, incidence, and mortality. The purpose of this study was to investigate how cervical cancer stage at diagnosis is associated with rurality and race/ethnicity.

**Methods:**

We analyzed 2010 through 2014 data from the Centers for Disease Control and Prevention’s National Program of Cancer Registries and the National Cancer Institute’s Surveillance, Epidemiology, and End Results Program. We compared cervical cancer frequency and age-adjusted incidence for each stage by county-level rurality and race/ethnicity.

**Results:**

There were 59,432 incident cases of cervical cancer reported from 2010 through 2014. The most common stage at diagnosis was localized (urban, 43.3%; rural 41.3%). Rural counties had higher incidence than urban counties for localized (rate ratio [RR] = 1.11; 95% confidence interval [CI], 1.07–1.15), regional (RR = 1.14; 95% CI, 1.10–1.19), and distant (RR = 1.12; 95% CI, 1.05–1.19) stage cervical cancer. Hispanic and non-Hispanic black women had higher incidence of regional and distant cervical cancer than non-Hispanic white women. Non-Hispanic white women in rural counties had higher incidence than those in urban counties at every stage. However, incidence for non-Hispanic white women was lower than for non-Hispanic black or Hispanic women.

**Conclusion:**

Rural counties had higher incidence of cervical cancer than urban counties at every stage. However, the association of rural residence with incidence varied by race/ethnicity.

**Summary**

**What is already known on this topic?**

Race/ethnicity and rural residence are associated with poorer cervical cancer outcomes.

**What is added by this report?**

We examined the association of rurality with cervical cancer stage at diagnosis by race/ethnicity in the US Cancer Statistics dataset for 2010–2014. Black and Hispanic women had higher incidence of regional and distant stage cancer than white women overall. Rural residents had higher rates of cervical cancer than urban residents at every stage (localized, regional, distant, and unknown). The association of rural residence with incidence varied by race/ethnicity.

**What are the implications for public health practice?**

Findings support the need for continued efforts to provide and promote cervical cancer screening in rural areas and among minority women.

## Introduction

Disparities in cervical cancer incidence and mortality are pervasive in the United States, despite improvements in outcomes over time (1). Understanding these disparities and related social determinants of health is critical to improving care (2). Race, socioeconomic status, and region strongly influence cervical cancer outcomes (3–5). For example, black women have higher incidence and mortality rates and lower survival rates than white women. Racial/ethnic disparities may reflect differences in cervical cancer screening rates and outcomes that vary by socioeconomic status and access to quality care (6–8). Because regular and timely screening can detect preclinical cervical lesions and early stage cancer, access to screening services and follow-up of abnormal tests can affect stage at diagnosis and overall cervical cancer incidence (9).

Women in rural areas may experience barriers to optimal cervical cancer prevention, early detection, and treatment. Overall, cervical cancer incidence and mortality rates are higher in rural and nonmetropolitan areas than in metropolitan areas (10–12). Studies have demonstrated lower use of screening services in rural areas (13,14).

The objective of this study was to investigate the relationship between rurality and cervical cancer stage at diagnosis by analyzing data from population-based cancer registries that cover the US population. We hypothesized that women in rural areas would have higher incidence of cervical cancer, particularly advanced-stage cancer, and that the effect of rurality may vary by race/ethnicity.

## Methods

We analyzed cancer incidence data from the Centers for Disease Control and Prevention’s (CDC’s) National Program of Cancer Registries and the National Cancer Institute’s (NCI’s) Surveillance, Epidemiology, and End Results Program. These data are submitted annually to CDC and NCI and combined (15). We used data for cancers diagnosed between 2010 and 2014, the most recent 5 years of data available. Data from Nevada did not meet United States Cancer Statistics (USCS) publication criteria in 2011, and Kansas and Minnesota do not report county-level data necessary to determine rurality so data from these states were excluded. The remaining incidence data covered approximately 96.5% of the US population. We included new cases of primary invasive cervical cancer (*International Classification of Diseases for Oncology, 3rd edition, *codes C53.0-C53.9) diagnosed among women aged 20 years or older (16). Cases diagnosed by death certificate only or autopsy (1.1%) were excluded.

Stage was classified using Summary Stage 2000 (https://seer.cancer.gov/tools/ssm), which characterizes cancers as localized (confined to primary site), regional (spread directly beyond primary site or to regional lymph nodes), distant (spread to other organs or remote lymph nodes), or unknown.

Rural–urban continuum codes (RUCC) from the US Department of Agriculture broadly describe counties as metropolitan or nonmetropolitan (www.ers.usda.gov/data-products/rural-urban-continuum-codes). RUCC 1 through 3 describe metropolitan counties with varying population sizes (≥1,000,000; 250,000– <1,000,000; and <250,000). Nonmetropolitan counties (RUCC 4–9) are further divided by urbanization, population size (<2,500; 2,500–19,999; and ≥20,000), and adjacency to a metropolitan area. Because exploratory analyses indicated homogeneity within metropolitan and nonmetropolitan categories, data were analyzed by 2 major groups: “urban” (RUCC 1–3) and “rural” (RUCC 4–9), similar to methods in other studies (10,17–19).

Data were analyzed for 4 mutually exclusive racial/ethnic groups: 1) non-Hispanic white; 2) non-Hispanic black; 3) non-Hispanic American Indian/Alaska Native, Asian/Pacific Islander, unspecified race, and other race and unknown (non-Hispanic other); and 4) Hispanic. Information about race and Hispanic ethnicity was collected separately. To reduce ethnic misclassification, the dataset uses the North American Association of Central Cancer Registries (NAACCR) Hispanic Identification Algorithm to assign Hispanic ethnicity (www.naaccr.org/wp-content/uploads/2016/11/NHIA_v2_2_1_09122011.pdf).

We used SEER*Stat version 8.3.4 (National Cancer Institute) to calculate average annual incidence for 2010 through 2014 per 100,000 women for each stage, by race/ethnicity and county type. Rates were age-adjusted by the direct method to the 2000 US standard population, with 95% confidence intervals (CIs) calculated as modified gamma intervals. This information can be used to assess whether the point estimate of one group falls within the confidence interval of the point estimate of another. Rates based on fewer than 16 cases may have poor reliability and are not presented. 

## Results

From 2010 through 2014, 59,432 incident cases of cervical cancer were reported (Table 1). Of these, most were diagnosed in urban counties (84.5% vs 15.5% in rural counties). Most cases were diagnosed among non-Hispanic (NH) white women (60.8%) followed by Hispanic (16.9%), NH black (15.7%), and NH other (6.6%) women. Localized cases were most commonly diagnosed for all groups except NH black women, for which regional stage predominated.

**Table 1:** Age-Adjusted Cervical Cancer Incidence (N = 59,432)^a^ and Rate Ratios of All Stages, by Race/Ethnicity and Rurality, US Cancer Statistics 2010–2014^b^

Cancer Stage	Race/Ethnicity				Rurality^c^		Total
	NH White	NH Black	Hispanic	NH Other^d^	Urban	Rural	
Total No. (%)	36,144 (60.8)	9,359 (15.7)	10,024 (16.9)	3,905 (6.6)	50,205 (84.5)	9,227 (15.5)	59,432
**Localized**							
No. (%)	16,080 (44.5)	3,384 (36.2)	4,412 (44.0)	1,688 (43.2)	21,757 (43.3)	3,807 (41.3)	25,564 (43.0)
Rate (95% CI)	4.7 (4.6–4.8)	4.8 (4.6–4.9)	5.6 (5.4–5.8)	4.5 (4.3–4.7)	4.7 (4.6–4.7)	5.2 (5.0–5.4)	—
Rate ratio (95% CI)	1 [Ref]	1.01 (0.97–1.05)	1.19 (1.15–1.23)	0.96 (0.91–1.01)	1 [Ref]	1.11 (1.07–1.15)	—
**Regional**							
No. (%)	12,603 (34.9)	3,719 (39.7)	3,697 (36.9)	1,369 (35.1)	17,981 (35.8)	3,407 (36.9)	21,388 (36.0)
Rate (95% CI)	3.2 (3.2–3.3)	5.2 (5.0–5.4)	5.1 (4.9–5.2)	3.8 (3.6–4.0)	3.6 (3.5–3.7)	4.1 (4.0–4.3)	—
Rate ratio (95% CI)	1 [Ref]	1.62 (1.56–1.68)	1.57 (1.51–1.63)	1.17 (1.10–1.24)	1 [Ref]	1.14 (1.10–1.19)	—
**Distant**							
No. (%)	5,496 (15.2)	1,637 (17.5)	1,266 (12.6)	475 (12.2)	7,434 (14.8)	1,440 (15.6)	8,874 (14.9)
Rate (95% CI)	1.3 (1.3–1.3)	2.3 (2.1–2.4)	1.8 (1.7–1.9)	1.3 (1.2–1.4)	1.4 (1.4–1.5)	1.6 (1.5–1.7)	—
Rate ratio (95% CI)	1 [Ref]	1.73 (1.63–1.83)	1.39 (1.30–1.48)	1.01 (0.92–1.11)	1 [Ref]	1.12 (1.05–1.19)	—
**Unknown**							
No. (%)	1,965 (5.4)	619 (6.6)	649 (6.5)	373 (9.6)	3,033 (6.0)	573 (6.2)	3,606 (6.1)
Rate (95% CI)	0.5 (0.5–0.5)	0.9 (0.8–0.9)	0.9 (0.8–1.0)	1.0 (0.9–1.1)	0.6 (0.6–0.6)	0.7 (0.6–0.7)	—
Rate ratio (95% CI)	1 [Ref]	1.75 (1.60–1.92)	1.83 (1.66–2.00)	2.04 (1.82–2.28)	1 [Ref]	1.13 (1.02–1.24)	—

Abbreviations: — , not applicable; CDC, Centers for Disease Control and Prevention; CI, confidence interval; NH, non-Hispanic; RUCC, rural–urban continuum codes; USDA, US Department of Agriculture.

a Rates are per 100,000 women and are age-adjusted to the 2000 US standard population (19 age groups, Census P25–1130).

b Data are from population-based registries that participate in CDC’s National Program of Cancer Registries and/or the National Cancer Institute’s Surveillance, Epidemiology, and End Results Program and meet high-quality data criteria. Data from Nevada did not meet criteria in 2011, and Kansas and Minnesota do not report county-level data, so were excluded. The remaining registries cover approximately 96.5% of the US population.

c Urban defined as USDA RUCC 1–3 (metropolitan counties with population sizes ≥1 million; 250,000– <1,000,000; and <250,000). Rural defined as RUCC 4–9 (nonmetropolitan counties with population sizes ≥20,000; 2,500–19,999; and <2,500).

d NH other includes non-Hispanic American Indian/Alaska Native, Asian/Pacific Islander, unspecified race, and other race and unknown.

Rural counties had higher age-adjusted incidence of localized, regional, and distant cervical cancer than urban counties (Table 1). Compared with NH white women, Hispanic women had higher incidence of cervical cancer for any stage. NH black women also had higher rates of regional and distant stage cancer, although rates of localized cancer were comparable to those of NH white women. NH black, NH other, and Hispanic women were more likely than white women to have unknown stage of disease.

Differences in incidence between urban and rural counties varied by race/ethnicity (Table 2). Among Hispanic women, no difference was observed for localized, regional, or distant stage cancer. NH black women in rural counties had higher incidence of regional cervical cancer than NH black women in urban counties. NH white women in rural counties had higher incidence than NH white women in urban counties for every stage cervical cancer. This effect was larger for regional than localized stage. For all racial/ethnic groups, rates of unknown stage cervical cancer were higher in rural than urban counties.

**Table 2:** Age-Adjusted Cervical Cancer Incidence (N = 59,432)^a^ and Rate Ratios of All Stages, by Rurality^b^ for Each Race/Ethnicity, US Cancer Statistics 2010–2014^c^

Stage	Race/Ethnicity							
	NH White		NH Black		Hispanic		NH Other^d^	
	Urban	Rural	Urban	Rural	Urban	Rural	Urban	Rural
**Localized**								
Rate (95% CI)	4.6 (4.5–4.7)	5.2 (5.0–5.4)	4.7 (4.6–4.9)	5.0 (4.5–5.6)	5.6 (5.4–5.8)	5.2 (4.6–5.9)	4.4 (4.2–4.6)	6.4 (5.4–7.5)
Rate ratio (95% CI)	1.13 (1.08–1.18)		1.05 (0.93–1.19)		0.93 (0.81–1.06)		1.46 (1.23–1.73)	
**Regional**								
Rate (95% CI)	3.1 (3.0–3.1)	3.9 (3.7–4.0)	5.1 (4.9–5.3)	6.2 (5.6–6.8)	5.1 (4.9–5.3)	4.6 (4.0–5.3)	3.7 (3.5–3.9)	4.7 (3.9–5.6)
Rate ratio (95% CI)	1.26 (1.21–1.32)		1.21 (1.09–1.34)		0.91 (0.78–1.05)		1.27 (1.04–1.54)	
**Distant**								
Rate (95% CI)	1.3 (1.2–1.3)	1.5 (1.4–1.6)	2.2 (2.1–2.4)	2.5 (2.1–2.9)	1.8 (1.7–1.9)	2.0 (1.5–2.4)	1.0 (0.9–1.1)	1.5 (1.0–2.1)
Rate ratio (95% CI)	1.21 (1.13–1.30)		1.11 (0.94–1.31)		1.08 (0.85–1.37)		1.40 (1.00–1.91)	
**Unknown**								
Rate (95% CI)	0.5 (0.5–0.5)	0.6 (0.5–0.7)	0.8 (0.8–0.9)	1.2 (0.9–1.5)	0.9 (0.8–1.0)	1.2 (0.9–1.7)	1.0 (0.9–1.1)	1.5 (1.0–2.1)
Rate ratio (95% CI)	1.23 (1.08–1.37)		1.38 (1.08–1.76)		1.40 (1.02–1.88)		1.52 (1.04–2.15)	

Abbreviations: — , not applicable; CDC, Centers for Disease Control and Prevention; CI, confidence interval; NH, non-Hispanic; RUCC, rural–urban continuum codes; USDA, US Department of Agriculture.

a Rates are per 100,000 women and are age-adjusted to the 2000 US standard population (19 age groups – Census P25–1130).

b Urban defined as USDA RUCC 1–3 (metropolitan counties with population sizes ≥1,000,000; 250,000–<1,000,000; and <250,000). Rural defined as RUCC 4–9 (nonmetropolitan counties with population sizes ≥20,000; 2,500–19,999; and <2,500).

c Data are from population-based registries that participate in CDC’s National Program of Cancer Registries and/or the National Cancer Institute’s Surveillance, Epidemiology, and End Results Program and meet high-quality data criteria. Data from Nevada did not meet criteria in 2011, and Kansas and Minnesota do not report county-level data, so were excluded. The remaining registries cover approximately 96.5% of the US population.

d NH other includes non-Hispanic American Indian/Alaska Native, Asian/Pacific Islander, unspecified race, and other race and unknown.

## Discussion

From 2010 through 2014, incidence of cervical cancer was higher in rural than urban counties, and this effect was similar for localized, regional, and distant stage cancer. However, the effect of rurality varied by race/ethnicity. White women had higher incidence of cervical cancer in rural counties for any stage. This pattern was not observed for black and Hispanic women, though these women had higher incidence of regional and distant cervical cancer than white women overall. Increases in these rates for black and Hispanic women appeared to be present among both urban and rural residents. Higher rates of localized disease for Hispanic women compared with white women overall may be due to the rates among urban women.

These results support the need for continued efforts to provide and promote cervical cancer screening in rural areas and among minority women. Screening can reduce cervical cancer mortality rates, especially from regional- and distant-stage cancer that have a poor prognosis (9,20). The National Breast and Cervical Cancer Early Detection Program has provided cervical cancer screening services to low-income, uninsured women for more than 20 years. However, the program reaches only a small proportion of eligible women, and many underserved women remain unscreened (21). In addition to detecting cancers earlier, screening can also reduce cervical cancer incidence through the detection and treatment of precancerous lesions. However, women in rural areas, who may have limited access to health care, are less likely to be up-to-date with cervical cancer screening (6,22). Non-Hispanic black and Hispanic women also had higher incidence of regional- and distant-stage disease than non-Hispanic white women, which is consistent with the higher overall cervical cancer incidence reported previously (5,8). Efforts to increase screening use among these women may help reduce these disparities.

No significant difference was found in localized, regional, or distant stage disease between rural and urban Hispanic women. Nuño et al suggested that Hispanic women in rural areas may have lower screening rates than Hispanic women in urban areas in the southwestern United States (23). Although we did not find differences between rural and urban Hispanic women, for both of these groups rates for each stage were higher than those for white women, with the exception of localized disease for which rates were similar between rural white and Hispanic women. Additional investigation may help elucidate why rates are higher for rural, black, and Hispanic women and may help inform efforts to address barriers for these groups.

We found that non-Hispanic black and Hispanic women were more likely than white women to have unknown stage of disease, consistent with other studies of cancer stage (24–26). Unknown stage was also more likely among rural compared with urban women, both overall and within each racial/ethnic group. The increased likelihood of unknown stage disease among rural residents is consistent with some literature on cancer but not all (26,27). Possible contributing factors include lower rates of insurance coverage (24) and less access to comprehensive diagnostic or treatment services (27). The reasons for higher rates of unstaged disease among rural and minority women may warrant further investigation, because unstaged cancer has been associated with worse survival than earlier stage cancers (25,28)

Our study has strengths and limitations. We used high-quality registry data that cover over 96% of the US population. Analyses based on the population better reflect national patterns than sample-based estimates. The county rurality codes have been used widely to demonstrate geographic differences (10,17–19). However, the measure of rurality based on county data offers less granularity than do census-tract data. Collapsing a multilevel variable into 2 categories may mask differences among subgroups within each category. However, the small numbers in some strata limited us from increasing the number of classification groups. Future efforts to build on these findings with more detailed information about rurality is important. We did not correct for hysterectomy, because cancer registries do not collect this information. Failure to adjust for hysterectomy status can lead to underestimates of cervical cancer incidence, especially among black women (5,29). Confounding may have occurred if the prevalence of hysterectomy differed between rural and urban counties.

Some factors that may have contributed to observed differences, such as cervical cancer screening history, were not available in our data set. Differences in incidence or in rates of later stage disease may reflect differences in screening use across groups. Hispanic women were less likely than non-Hispanic women to be recently screened for cervical cancer in 2015 (30). We observed higher cervical cancer incidence, including higher rates of regional or distant disease, among Hispanic women compared with non-Hispanic white women. We did not have information about availability of or proximity to screening and diagnostic services (14). Delays in receipt of timely diagnosis after an abnormal screening test could contribute to later stage at diagnosis.

In conclusion, higher cervical cancer incidence among women in rural areas highlights missed opportunities to implement evidence-based interventions to promote cancer screening and increase screening use in rural communities. Multicomponent interventions designed to increase community demand for or access to cancer screening and to increase provider delivery of screening services can increase cervical cancer screening use (www.thecommunityguide.org/). Consideration is needed of barriers, including those which may be relevant in rural populations (eg, increased travel distances). Ensuring access to screening and diagnostic services for rural women is critical to reduce cervical cancer disparities.

## References

[1] Jemal A , Ward EM , Johnson CJ , Cronin KA , Ma J , Ryerson B , Annual report to the nation on the status of cancer, 1975–2014, featuring survival. J Natl Cancer Inst 2017;109(9). 10.1093/jnci/djx030 28376154 PMC5409140

[2] Committee on Health Care for Underserved Women. ACOG Committee Opinion No. 729: importance of social determinants of health and cultural awareness in the delivery of reproductive health care. Obstet Gynecol 2018;131(1):e43–8. 10.1097/AOG.0000000000002459 29266079

[3] Beavis AL , Gravitt PE , Rositch AF . Hysterectomy-corrected cervical cancer mortality rates reveal a larger racial disparity in the United States. Cancer 2017;123(6):1044–50. 10.1002/cncr.30507 28112816

[4] Benard VB , Watson M , Saraiya M , Harewood R , Townsend JS , Stroup AM , Cervical cancer survival in the United States by race and stage (2001-2009): findings from the CONCORD-2 study. Cancer 2017;123(Suppl 24):5119–37. 10.1002/cncr.30906 29205300 PMC6386458

[5] White MC , Shoemaker ML , Benard VB . Cervical cancer screening and incidence by age: unmet needs near and after the stopping age for screening. Am J Prev Med 2017;53(3):392–5. 10.1016/j.amepre.2017.02.024 28473240 PMC5821231

[6] Datta GD , Colditz GA , Kawachi I , Subramanian SV , Palmer JR , Rosenberg L . Individual-, neighborhood-, and state-level socioeconomic predictors of cervical carcinoma screening among U.S. black women: a multilevel analysis. Cancer 2006;106(3):664–9. 10.1002/cncr.21660 16378349

[7] Saghari S , Ghamsary M , Marie-Mitchell A , Oda K , Morgan JW . Sociodemographic predictors of delayed- versus early-stage cervical cancer in California. Ann Epidemiol 2015;25(4):250–5. 10.1016/j.annepidem.2015.01.008 25794765

[8] Benard VB , Thomas CC , King J , Massetti GM , Doria-Rose VP , Saraiya M ; Centers for Disease Control and Prevention (CDC). Vital signs: cervical cancer incidence, mortality, and screening - United States, 2007-2012. MMWR Morb Mortal Wkly Rep 2014;63(44):1004–9. 25375072 PMC5779486

[9] Landy R , Pesola F , Castañón A , Sasieni P . Impact of cervical screening on cervical cancer mortality: estimation using stage-specific results from a nested case-control study. Br J Cancer 2016;115(9):1140–6. 10.1038/bjc.2016.290 27632376 PMC5117785

[10] Henley SJ , Anderson RN , Thomas CC , Massetti GM , Peaker B , Richardson LC . Invasive cancer incidence, 2004–2013, and deaths, 2006–2015, in nonmetropolitan and metropolitan counties — United States. MMWR Surveill Summ 2017;66(14):1–13. 10.15585/mmwr.ss6614a1 28683054 PMC5879727

[11] Singh GK . Rural-urban trends and patterns in cervical cancer mortality, incidence, stage, and survival in the United States, 1950-2008. J Community Health 2012;37(1):217–23. 10.1007/s10900-011-9439-6 21773819

[12] Moss JL , Liu B , Feuer EJ . Urban/rural differences in breast and cervical cancer incidence: the mediating roles of socioeconomic status and provider density. Womens Health Issues 2017;27(6):683–91. 10.1016/j.whi.2017.09.008 29108988 PMC5694385

[13] Caldwell JT , Ford CL , Wallace SP , Wang MC , Takahashi LM . Intersection of living in a rural versus urban area and race/ethnicity in explaining access to health care in the United States. Am J Public Health 2016;106(8):1463–9. 10.2105/AJPH.2016.303212 27310341 PMC4940644

[14] McDonald YJ , Goldberg DW , Scarinci IC , Castle PE , Cuzick J , Robertson M , Health service accessibility and risk in cervical cancer prevention: comparing rural versus nonrural residence in New Mexico. J Rural Health 2017;33(4):382–92. 10.1111/jrh.12202 27557124 PMC5939944

[15] National Program of Cancer Registries. Surveillance, Epidemiology and End Results SEER*Stat Database: USCS Incidence Analytic Database — 1998–2014. Atlanta (GA): US Department of Health and Human Services, Centers for Disease Control and Prevention.

[16] Fritz A , Percy C , Jack A , Shanmugarathnam K , Sobin L , Parkin D , International classification of disease for oncology. 3rd ed. Geneva (CH): World Health Organization; 2000.

[17] Modesitt SC , Huang B , Shelton BJ , Wyatt S . Endometrial cancer in Kentucky: the impact of age, smoking status, and rural residence. Gynecol Oncol 2006;103(1):300–6. 10.1016/j.ygyno.2006.03.009 16631234

[18] Zahnd WE , Hyon KS , Diaz-Sylvester P , Izadi SR , Colditz GA , Brard L . Rural-urban differences in surgical treatment, regional lymph node examination, and survival in endometrial cancer patients. Cancer Causes Control 2018;29(2):221–32. 10.1007/s10552-017-0998-4 29282582 PMC6311991

[19] Vanderboom CP , Madigan EA . Federal definitions of rurality and the impact on nursing research. Res Nurs Health 2007;30(2):175–84. 10.1002/nur.20194 17380518

[20] Ekwueme DU , Uzunangelov VJ , Hoerger TJ , Miller JW , Saraiya M , Benard VB , Impact of the National Breast and Cervical Cancer Early Detection Program on cervical cancer mortality among uninsured low-income women in the U.S., 1991-2007. Am J Prev Med 2014;47(3):300–8. 10.1016/j.amepre.2014.05.016 25015564

[21] White MC , Wong FL . Preventing premature deaths from breast and cervical cancer among underserved women in the United States: insights gained from a national cancer screening program. Cancer Causes Control 2015;26(5):805–9. 10.1007/s10552-015-0541-4 25783456 PMC4431943

[22] Horner-Johnson W , Dobbertin K , Iezzoni LI . Disparities in receipt of breast and cervical cancer screening for rural women age 18 to 64 with disabilities. Womens Health Issues 2015;25(3):246–53. 10.1016/j.whi.2015.02.004 25864023

[23] Nuño T , Gerald JK , Harris R , Martinez ME , Estrada A , García F . Comparison of breast and cervical cancer screening utilization among rural and urban Hispanic and American Indian women in the Southwestern United States. Cancer Causes Control 2012;23(8):1333–41. 10.1007/s10552-012-0012-0 22710745

[24] Merrill RM , Sloan A , Anderson AE , Ryker K . Unstaged cancer in the United States: a population-based study. BMC Cancer 2011;11(1):402. 10.1186/1471-2407-11-402 21936934 PMC3188514

[25] Worthington JL , Koroukian SM , Cooper GS . Examining the characteristics of unstaged colon and rectal cancer cases. Cancer Detect Prev 2008;32(3):251–8. 10.1016/j.cdp.2008.08.006 18804920

[26] Hsieh MC , Yu Q , Wu XC , Wohler B , Fan Y , Qiao B , Evaluating factors associated with unknown SEER Summary Stage 2000 derived from collaborative stage at central registry level. J Registry Manag 2012;39(3):101–6. 23443453 PMC5861716

[27] Lengerich EJ , Tucker TC , Powell RK , Colsher P , Lehman E , Ward AJ , Cancer incidence in Kentucky, Pennsylvania, and West Virginia: disparities in Appalachia. J Rural Health 2005;21(1):39–47. 10.1111/j.1748-0361.2005.tb00060.x 15667008

[28] Merrill RM , Henson DE , Barnes M . Conditional survival among patients with carcinoma of the lung. Chest 1999;116(3):697–703. 10.1378/chest.116.3.697 10492274

[29] Dalton HJ , Farley JH . Racial disparities in cervical cancer: Worse than we thought. Cancer 2017;123(6):915–6. 10.1002/cncr.30501 28112811

[30] White A , Thompson TD , White MC , Sabatino SA , de Moor J , Doria-Rose PV , Cancer screening test use — United States, 2015. MMWR Morb Mortal Wkly Rep 2017;66(8):201–6. 10.15585/mmwr.mm6608a1 28253225 PMC5657895

